# Emergency department non-invasive cardiac output study (EDNICO): an accuracy study

**DOI:** 10.1186/s13049-020-0704-5

**Published:** 2020-01-31

**Authors:** David McGregor, Shrey Sharma, Saksham Gupta, Shanaz Ahmed, Tim Harris

**Affiliations:** 1 Queen Mary University London and Barts Health NHS Trust, London, UK; 2 University of Western Australia School of Medicine and Pharmacology, Perth, Australia; 3 Emergency Department Research Group, Royal London Hospital, London, UK

**Keywords:** Fluid responsiveness, Stroke volume, Ultrasound, Bioreactance, Plethysmography, Sepsis

## Abstract

**Background:**

There is little published data investigating non-invasive cardiac output monitoring in the emergency department (ED). We assess here the accuracy of five non-invasive methods in detecting fluid responsiveness in the ED: (1) common carotid artery blood flow, (2) suprasternal aortic Doppler, (3) bioreactance, (4) plethysmography with digital vascular unloading method, and (5) inferior vena cava collapsibility index. Left ventricular outflow tract echocardiography derived velocity time integral is the reference standard. This follows an assessment of feasibility and repeatability of these methods in the same cohort of ED patients.

**Methods:**

This is a prospective observational study of non-invasive methods for assessing fluid responsiveness in the ED. Participants were non-ventilated ED adult patients requiring intravenous fluid resuscitation. Sensitivity and specificity of each method in determining the fluid responsiveness status of participants is determined in comparison to the reference standard.

**Results:**

Thirty-three patient data sets were included for analysis. The specificity and sensitivity to detect fluid responders was 46.2 and 45% for common carotid artery blood flow (CCABF), 61.5 and 63.2% for suprasternal artery Doppler (SSAD), 46.2 and 50% for bioreactance, 50 and 41.2% for plethysmography vascular unloading technique (PVUT), and 63.6 and 47.4% for inferior vena cava collapsibility index (IVCCI), respectively. Analysis of agreement with Cohen’s Kappa − 0.08 for CCABF, 0.24 for SSAD, − 0.04 for bioreactance, − 0.08 for PVUT, and 0.1 for IVCCI.

**Conclusion:**

In this study, non-invasive methods were not found to reliably identify fluid responders. Non-invasive methods of identifying fluid responders are likely to play a key role in improving patient outcome in the ED in fluid depleted states such as sepsis. These results have implications for future studies assessing the accuracy of such methods.

## Background

The aim of intravenous fluid therapy is to increase cardiac output and therefore to increase oxygen delivery to hypo-perfused organs. Resuscitation with inadequate intravenous fluid may risk inadequate organ oxygen delivery. Over resuscitation may precipitate iatrogenic tissue oedema leading to compartment syndromes and decreased oxygen delivery, with consequent increases in mortality [1–3]. Early-stage fluid resuscitation is commonly guided by physiological parameters such as capillary refill time, pulse rate, and blood pressure. Biochemical parameters such as lactate and acid-base assessment may also be used to guide fluid doses [4]. Neither approach offers a sensitive or specific proxy for organ perfusion, so in-precisely guiding fluid dosing [5–9].

Fluid responsiveness is commonly defined as a stroke volume increase of at least 10% following a fluid bolus of 200-500mls delivered over 10–15 min [10]. Around one to two thirds of emergency department (ED) patients thought to require fluid resuscitation are not fluid responsive and risk harm from aggressive fluid administration [11]. Identifying which patients respond to fluids (and continue to do so) has the potential to individualise fluid delivery and improve outcomes [12]. In intensive care units, invasive monitoring of cardiac output is frequently used to guide fluid administration, with methods including pulmonary artery catheterisation (PAC), arterial pulse pressure analysis or oesophageal Doppler [13]. However, these methods are invasive and unsuitable for routine monitoring in the ED [14, 15].

Non-invasive cardiac output monitoring methods are emerging in the ED and in the pre-hospital environment [16, 17]. These methods include common carotid artery blood flow monitoring (CCABF) [18], suprasternal aortic Doppler (SSAD) [19, 20], plethysmography using the vascular unloading technique (PVUT) [21, 22], and thoracic bioreactance [23–25]. Inferior vena cava collapsibility index (IVCCI), while not measuring cardiac output, has been studied as an indicator of fluid responsiveness [25–27]. Stroke volume measured by left ventricular outflow tract velocity time integral (LVOT VTI) [28, 29] is the most widely studied of these techniques, including validation against the PAC, which is widely referred to as the gold standard for cardiac output monitoring. LVOT VTI is widely accepted as a non-invasive method to assess stroke volume and cardiac output, and guide fluid therapy [30–35]. We thus arbitrarily assigned the LVOT VTI as reference standard in the current study whist being aware of its limitations in terms of operator experience/skill.

The clinical value of a diagnostic test is assessed by a multi-phase process which includes assessing its feasibility, repeatability, accuracy, impact on patient outcomes and cost. We have previously reported on the feasibility and repeatability of the above methods in these same cohort of ED patients [36]. We report here the diagnostic accuracy of these techniques determined by the sensitivity and specificity of each in identifying fluid responders as identified by LVOT VTI.

## Methods

### Study protocol

**Fig. 1:**
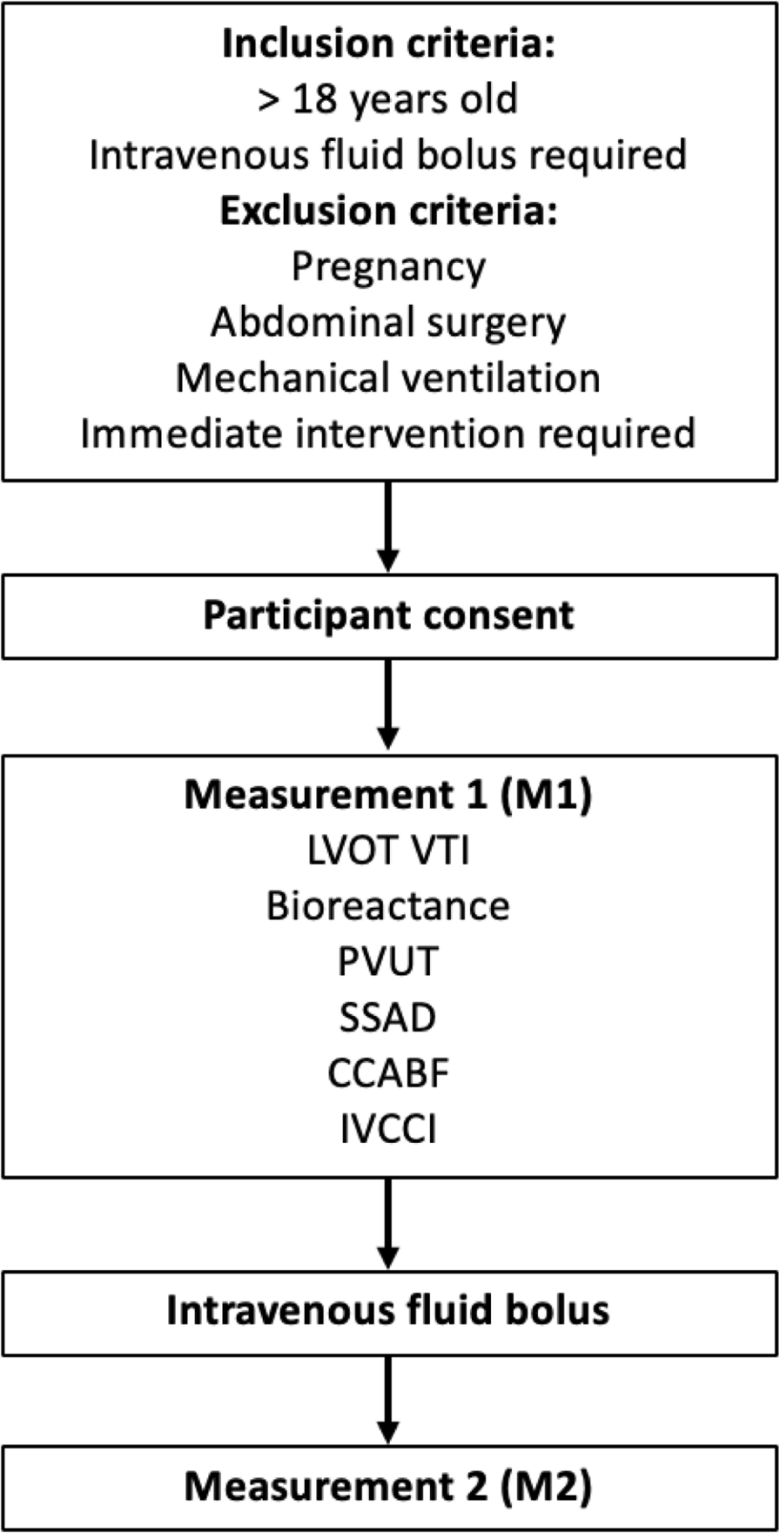
Study participant pathway. SV = stroke volume; CO = cardiac output; PVUT = plethysmography vascular unloading technique; CCABF = common carotid artery blood flow; IVCCI = inferior vena cava collapsibility index; LVOT VTI = left ventricular outflow tract velocity time integral; SSAD = suprasternal aortic Doppler. Immediate intervention required signifies here a systolic blood pressure < 80 mmHg including traumatic or cardiogenic shock, and ventricular or supraventricular tachycardia)

The study was a prospective observational diagnostic accuracy study. The Standards for Reporting of Diagnostic Accuracy Studies (STARD) were followed [37]. Accuracy was determined by sensitivity and specificity of each method in identifying fluid responders as determined by the assigned reference standard LVOT VTI. Participants were placed in a semi-recumbent position at 30 degrees on a trolley. The stroke volume was simultaneously measured by LVOT VTI, CCABF, bioreactance, and PVUT (measurement round 1 - M1) (Fig. 1). The IVCCI was also measured. A fluid bolus of 250–500 mls of crystalloid was then delivered over 15 min or less. A measurement margin of error of up to 10% in the estimated volume delivered, and up to 1 min in delivering the fluid bolus, was deemed acceptable. A post-fluid measurement round with all six methods was then conducted (M2). Fluid responsiveness was defined as a stroke volume increase of 10% or more from M1 to M2 by LVOT VTI using Dinh et al’s method [38]. Stolz et al’s method was used to measure CCABF [39]; an CCABF increase of 10% identified fluid responsiveness in this index test. The Fremantle criteria were used to measure stroke volume by SSAD [40]. IVCCI was measured in B mode with the minimal and maximal IVC diameters during respiration measured 1 cm distal to the hepatic-caval junction or 2-3 cm distal to the atrial-caval junction as guided by previous studies [26, 27, 41, 42]. IVCCI was calculated with the following formula:

$$ \frac{IVC\  diameter\ (expiration)- IVC\  diameter\ (inspiration)}{IVC\  diameter\ (expiration)} $$

Stroke volume by PVUT and bioreactance was measured following the manufacturer’s instructions.

### Participants

All participants recruited for the previously reported feasibility and repeatability analysis were also considered for inclusion in the present method accuracy analysis [36]. Briefly, potential participants were screened for inclusion and exclusion criteria by the clinical team on arrival to the ED to minimise selection bias. Patients requiring immediate intervention were excluded (systolic blood pressure < 80 mmHg including traumatic or cardiogenic shock, and ventricular or supraventricular tachycardia). All eligible patients were then referred to the research team for consent. All patients attending the ED during the study period during the hours of 09:00 to 20:00 Monday to Friday between August to October were eligible for recruitment. Patients having received fluids in a pre-hospital setting were not excluded from the study.

### Equipment

LVOT VTI was measured with a uSmart 3300 ultrasound system (Terason, Burlington, MA, USA). Carotid Doppler traces were assessed by a Sonosite EDGE (Sonosite, Bothwell, WA, USA). Suprasternal aortic Doppler traces were obtained using the USCOM-1A (Pty Ltd., Coffs Harbour, NSW, Australia). PVUT was assessed with a LiDCO continuous non-invasive arterial pressure device (LiDCO plus and CNAP, LiDCO Ltd., London, UK). Bioreactance was assessed with a Cheetah Medical device (Cheetah Medical, Portland, OR, USA).

### Operator training

Three operators (DM, SG, SS) with no prior ultrasound experience were trained to operate all six non-invasive monitoring methods during the same pre-study training programme as previously described [36]. Briefly, this consisted of the standard UK Level 1 ultrasound course followed by 50 measurements for all four ultrasound methods on volunteers. Competency for each method was confirmed through triggered assessment by the regional Royal College of Emergency Medicine lead for ultrasound training. Bioreactance and PVUT training was provided by the respective manufacturers for 2 h each. This training programme ensured that operators’ experience on all methods was identical and achievable by junior residents and ED nurses. Interobserver reliability for LVOT VTI amongst operators was satisfactory and reported in a separate study [43].

### Statistical analysis

Measurement data were collected onto a REDCap database (Vanderbilt University, Nashville, TN, USA) and analysed with SPSS v24 (IBM, New York City, NY, USA) and STATA 15.1 (StataCorp, College Station, TX, US). *P* < 0.05 (two-tailed) was considered statistically significant. To compare proportions of fluid responders with an expected sensitivity of at least 0.80 for all index tests and a minimum acceptable lower confidence limit of 0.50, an a priori minimum sample size of 28 was required [44]. Mann-Whitney, Chi square and Fisher exact tests were used where appropriate. Agreement between the reference standard and the index tests was also analysed with Cohen’s kappa (k) as described in previous studies of cardiac output/stroke volume assessment methods [45–47]. A kappa value of 1 shows perfect agreement between methods (0.8–1: strong agreement, 0.7–0.8: good agreement, 0.5–0.7: moderate agreement, < 0.5: poor agreement). Specificity and sensitivity values for each method were also calculated.

### Ethics, consent and permissions

This study was approved by the East of England Research Ethics Committee (REC reference 15/EE/0227; IRAS project ID 172012). Informed consent by each subject was required for participation in this study.

## Results

**Fig. 2:**
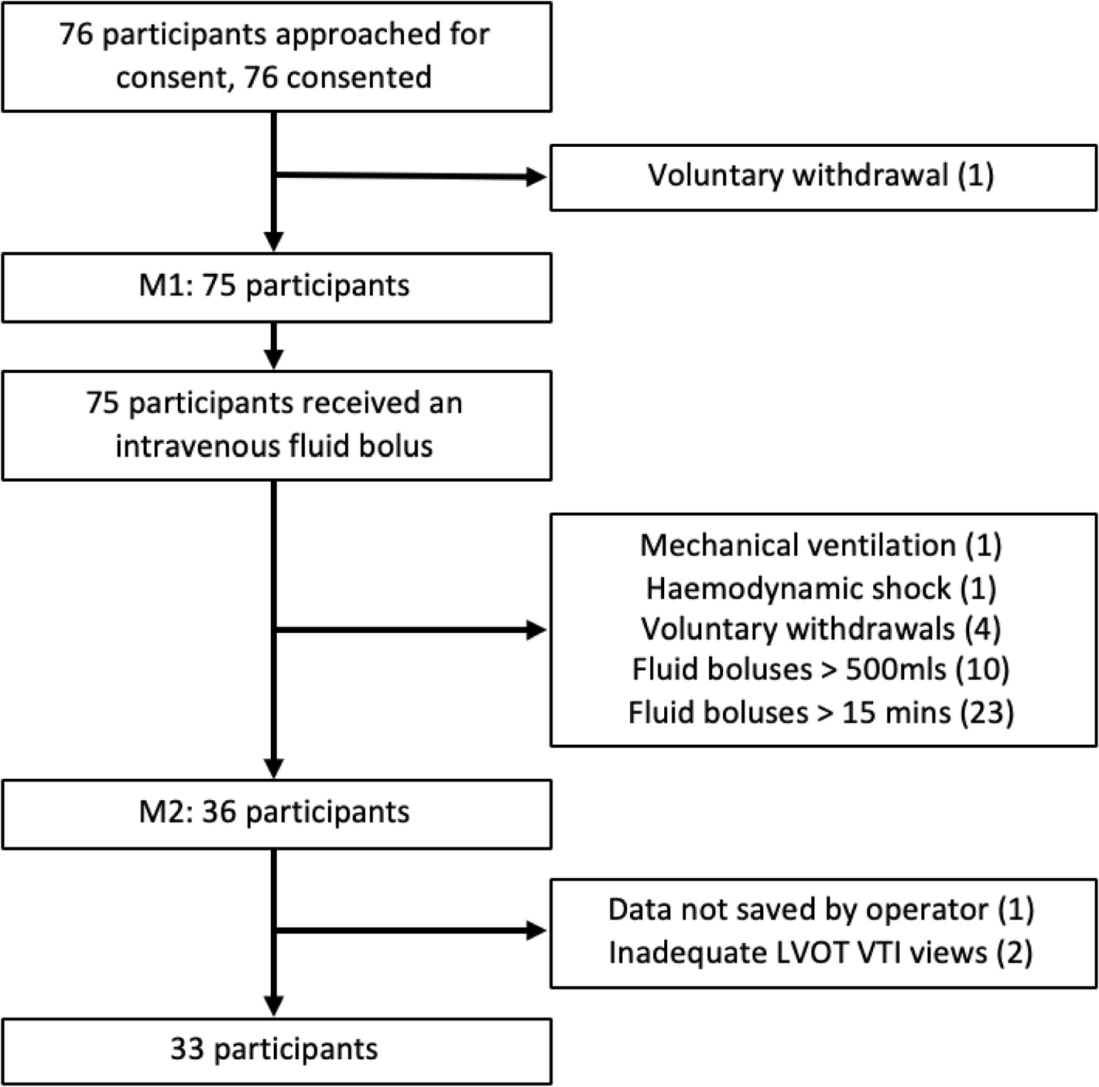
Study flowchart

Of 76 participants recruited in the primary study [36], 33 received 250-500mls of crystalloid over 15 min or less and were included for assessment of accuracy (Fig. 2). Reasons for exclusion included a prescribed change in fluid delivery rate by the clinical care team, interruption of fluid monitoring for patient transfer, and rate-limited fluid infusion equipment. 60.6% (20/33) of participants were identified as fluid responders (Table 1).

**Table 1:** Participant characteristics. Characteristics are displayed at baseline (prior to fluid bolus) and then compared between fluid responders (FRs) and non-responders (NRs). Data expressed in means and standard deviations. BMI: body mass index, MAP: mean arterial pressure, SBP: systolic blood pressure, DBP: diastolic blood pressure

	All participants (*n* = 33)	Fluid non-responders (*n* = 13)	Fluid responders (*n* = 20)	*p* value
Age	48.4 (21.8)	45.5 (24.1)	50.2 (20.6)	0.55
Sex (F:M)	16:17	11:9	5:8	0.35
BMI	25.7 (6.4)	25.2 (7.2)	26 (5.9)	0.73
MAP	91.3 (16.7)	94 (16.3)	89 (16.9)	0.33
SBP	126.3 (23.9)	132.7 (22.2)	122.1 (24.5)	0.22
DBP	69.36 (16.7)	74 (17.8)	66.3 (15.6)	0.20
Heart rate	93.5 (20.7)	97.6 (17.8)	90.9 (22.4)	0.37
Lactate	6 (11.1)	7.0 (14.8)	5.1 (6.8)	0.71
Fluid bolus (FB)	472.7 (83)	461.5 (86.9)	480 (81.7)	0.54
Duration of FB	11.5 (2)	11.9 (2.5)	11.2 (1.6)	0.36

**Fig. 3:**
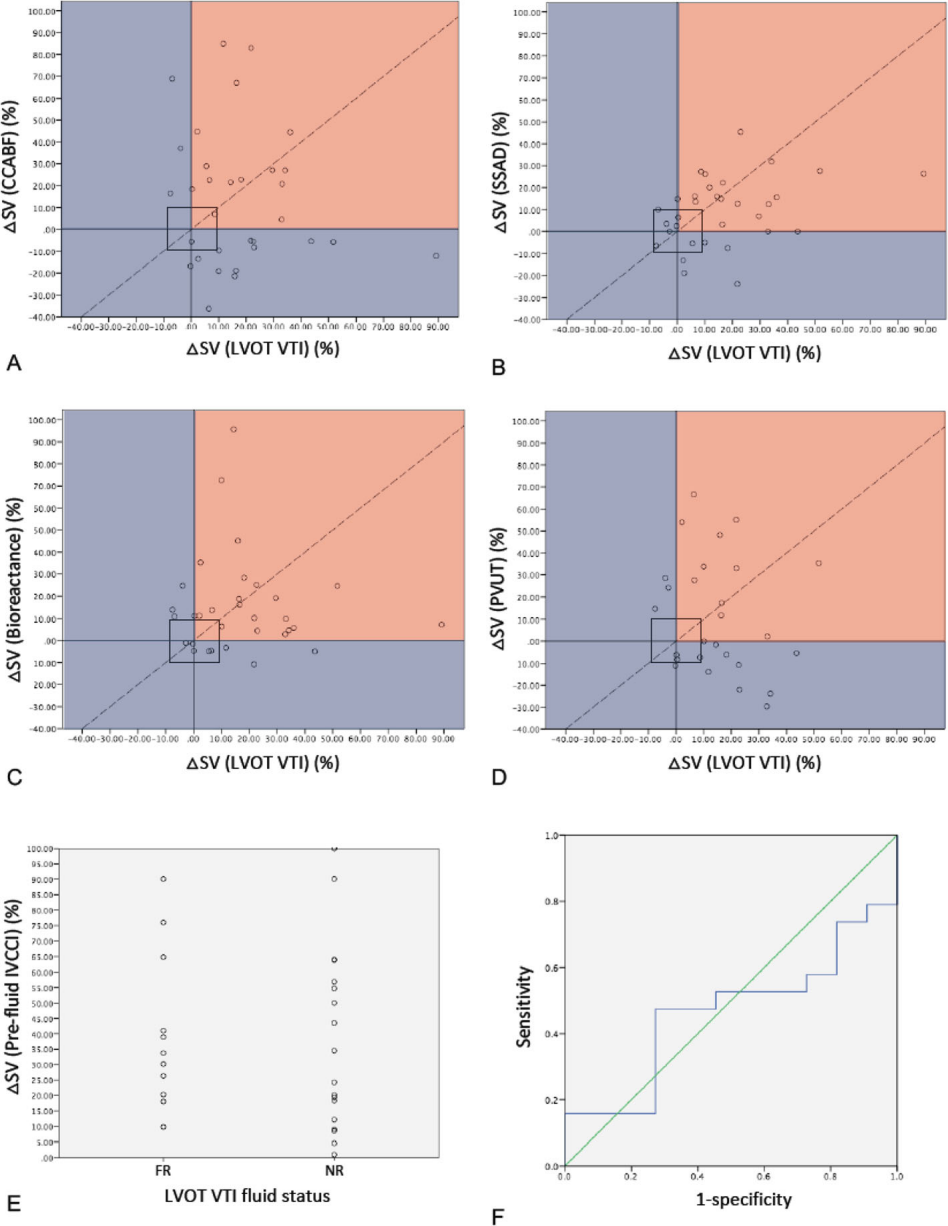
Accuracy of each method was assessed by its agreement with LVOT VTI (left ventricular outflow tract velocity time integral) in identifying fluid responders. Changes in stroke volume (ΔSV) after fluid challenge as determined by LVOT VTI are plotted against CCABF (common carotid artery blood flow) (**a**), SSAD (suprasternal aortic Doppler) (**b**), bioreactance (**c**), and PVUT (plethysmography vascular unloading technique) (**d**). A 10% exclusion zone is marked by a square in the concordance plots. Dots in the right upper quadrant (red area) indicate agreement between LVOT VTI and the test method in identifying fluid responders. IVCCI was assessed with a dot plot (**e**) which shows the IVCCI values of fluid responders (FR) on the left and fluid non-responders (NR) on the right. The receiver operating characteristic curve (**f**) had an area under the receiver operating curve (AUROC) of 0.464 (*p* = 0.747) [95% CI 0.264–0.675]

The measure of agreement between the reference standard (LVOT VTI) and test method to identify fluid responders was expressed as a kappa value (Table 2). A four-quadrant plot illustrates the concordance of directional change for each patient as measured by each test method as compared to LVOT VTI. An exclusion zone for small changes in stroke volume is applied to exclude changes of stroke volume of less than 10% (Fig. 3). To assess the accuracy of IVCCI in identifying fluid responders, the optimal IVCCI cut-off was 40%. A receiver operating characteristic (ROC) curve was plotted (Fig. 3f).

**Table 2:** Diagnostic accuracy of test methods. Agreement between LVOT VTI and test methods in identifying fluid responders is displayed using Cohen’s kappa values. The agreement was poor to null across the above four methods. FR: fluid responders, NR: fluid non-responders, LVOT VTI: left ventricular outflow tract velocity time integral, CCABF: common carotid artery blood flow, SSAD: suprasternal aortic Doppler, PVUT: plethysmography with vascular unloading technique, IVCCI: inferior vena cava collapsibility index

	LVOT VTI FR	LVOT VTI NR
Total	20	13
CCABF FR	9	7
CCABF NR	11	6
Specificity	46.2% (95% CI: 19.2–74.9%)	
Sensitivity	45% (95% CI: 23.1–68.5%)	
Kappa	−0.0839	
Total	19	13
SSAD FR	12	5
SSAD NR	7	8
Specificity	61.5% (95% CI: 31.6–86.1%)	
Sensitivity	63.2% (95% CI: 38.4–83.7%)	
Kappa	0.2411	
Total	20	13
Bioreactance FR	10	7
Bioreactance NR	10	6
Specificity	46.2% (95% CI:19.2–74.9%)	
Sensitivity	50% (95% CI: 27.2–72.8%)	
Kappa	−0.0370	
Total	17	12
PVUT FR	7	6
PVUT NR	10	6
Specificity	50% (95% CI: 21.1–78.9%)	
Sensitivity	41.2% (95% CI: 18.4–67.1%)	
Kappa	−0.0841	
Total	19	11
IVCCI FR	9	4
IVCCI NR	10	7
Specificity	63.6% (95% CI: 30.8–89.1%)	
Sensitivity	47.4% (95% CI: 24.4–71.1%)	
Kappa	0.0987

## Discussion

This study finds that non-invasive cardiac output methods have poor agreement in identifying fluid responders in spontaneously breathing ED patients.

We identified no studies defining the accuracy of bioreactance in assessing fluid responsiveness in spontaneously breathing ED patients. To date studies performed heterogenous results. Squara et al. reported good agreement between cardiac output as measured by bioreactance and PAC in 110 patients [24]. However, accuracy to identify fluid responders was poor when studied on a subset of 23 patients with an assortment of interventions (7 rapid infusion challenges, 6 dobutamine challenges, 6 high PEEP stops, and 4 adrenaline infusions). Marque et al. also compared bioreactance to PAC in a group 19 mechanically ventilated patients who underwent rapid fluid infusion or passive leg raise (PLR) [48]. Bioreactance was found to have a sensitivity of 91% and a specificity of 95% in identifying fluid responders (no confidence intervals reported). Similarly, Galarza et al. reported reasonable accuracy in identifying fluid responders amongst 32 ICU patients with a sensitivity of 92% (95% CI: 62–91%) and a specificity of 80% (95% CI: 56–94%) when using an upgraded bioreactance device against PAC [49]. However, Kupersztych-Hagege et al. reported that the AUROC for bioreactance when compared to PAC to identify fluid responders amongst 48 critically ill patients was not significantly different to 0.5 (*p* = 0.77; no confidence intervals reported) [50]. The study was criticised for its methodology [51, 52]. Similarly, in a cohort of 22 patients under anaesthesia prior to elective intra-abdominal surgery, Conway et al. reported a sensitivity of 75% and a specificity of 69% in identifying fluid responders against oesophageal Doppler monitoring (no confidence intervals reported) [53]. Lastly, De Pascale et al. reported a sensitivity of 80% (95% CI: 56.3–94.3) and specificity of 82.6% (95% CI: 68.6–92.2) in 21 patients under anaesthesia prior to elective pelvic surgery against oesophageal Doppler monitoring [54].

In the current study, PVUT showed no agreement with LVOT VTI in identifying fluid responders. Movement of the digit on which the cuff device is placed was found to delay calibration and interfere with PVUT measurements. Other studies have investigated PVUT in its accuracy in predicting fluid responsiveness through pulse pressure variation (PPV), rather than measuring stroke volume change. Biais et al. reported that in 35 patients under general anaesthesia, a baseline 15% PPV threshold measured through CNAP identified fluid responders with a sensitivity of 76% (95% CI: 53–92%) and a specificity of 93% (95% CI: 66–99%) [55]. Similarly, Monnet et al. reported a sensitvity of 82% (95% CI: 57–96%) and a specificity of 91% (95% CI: 71–99%) for CNAP-derived PPV against pulse pressure analysis in 39 critically ill mechanically ventilated patients [56].

Previous studies on CCABF were conducted on healthy volunteers [57, 58], ICU patients [18, 59–61], and peri-operative patients [62, 63]. To our knowledge no studies have investigated the accuracy of SSAD in identifying fluid responders in spontaneously breathing patients in any clinical setting. Marik et al’s retrospective data review of 34 ICU patients found that CCABF identified fluid responders, using PLR as a surrogate for fluid loading, with a sensitivity of 94% and a specificity of 86% (no confidence levels or AUROC reported) [18]. 19 (56%) participants were mechanically ventilated, reducing variations in intra-thoracic pressure compared to spontaneous breathing potentially improving CCABF accuracy. However, Roehrig et al’s study of 33 post-operative mechanically ventilated patients reported an AUROC of 0.54 (95% CI: 0.32–0.76) using PAC as a reference method. The authors suggest that cerebral blood flow autoregulation possibly explains why CCABF did not reflect cardiac output changes after PLR [63]. PLR is a low-cost, easily accessible, and well validated alternative to an intravenous fluid bolus to assess fluid responsiveness. However, the latter is still first line practice in many UK EDs, and was therefore selected as the method of choice in this study.

Although SSAD is reported to reliably detect a 7.5% blood volume loss in healthy volunteers few studies have investigated its accuracy to detect fluid responsiveness [64, 65]. A meta-analysis on SSAD accuracy in mechanically ventilated patients by Chong and Peyton found an overall cardiac output percentage error of 42.7% (95% CI: 38.5–46.9%) in comparison to PAC [66]. The authors hypothesized that inaccuracy arises from operators obtaining a suboptimal angle of incidence on the direction of aortic blood flow.

For IVCCI accuracy, a recent systematic review by Seccombe at al identified six studies conducted in spontaneously breathing patients with sepsis. The authors reported that the high level of heterogeneity amongst study designs prevented pooling of results [67]. The lack of studies conducted in non-ICU settings, where most patients with sepsis are managed, also contributed to poor generalisability of results. The heterogeneity of techniques in measuring IVCCI has been previously discussed [36]. Four studies used LVOT VTI as the reference method, one used bioreactance, and one used systolic blood pressure. The study of Lanspa et al. was the only one based in the ED. The authors reported an IVCCI sensitivity of 100%, a specificity of 67% (no CI reported), and an AUROC of 0.83 (95% CI: 0.58–1.00) to detect fluid responders amongst 14 patients using LVOT VTI as the reference standard [26].

Judicious intravenous fluid delivery plays an important role in increasing oxygen delivery to hypoperfused organs and avoiding fluid overload. As discussed above, few studies have investigated non-invasive methods to identify fluid responsive states in spontaneously breathing patients. This group represents the majority of patients with sepsis who receive intravenous fluid therapy and consequently requires further study. Overall, reviewed studies report low to moderate accuracy across all methods.

### Study limitations

This study has several limitations. A window of 15 min was specified in the protocol to allow all 6 measures to be taken in each round as patients generally do not tolerate more than three measures to be taken at once. Therefore, not all measures occurred simultaneously. This window of time could be sufficient for a physiological change of stroke volume to occur, especially in patients in which fluid is quickly re-distributed to extra-vascular compartments. This would increase the chances of a significant agreement between methods being missed when it actually exists (type II error). All measurements were taken as efficiently as possible to mitigate this effect. A second limitation was that the order with which methods were used during the measurement windows did not vary. The least restrictive methods were deployed first to minimise distress in patients. Anecdotally, bioreactance and PVUT were best tolerated, followed by IVCCI, CCABF, LVOT VTI and SSAD. There is little evidence available on duration of the effect of a fluid bolus in fluid depleted patients. It is reasonable to assume that this duration varies with the cause of the fluid depletion and its severity. Monnet and Teboul found that a PLR manoeuvre, during which the patient’s leg are raised by 45 degrees to increase venous return to the heart, induced its maximal preload effect within 90 s in critically ill patients with a subsequent return to baseline [68]. It is uncertain how long an increased preload, either by PLR or fluid bolus, is maintained for. Lastly, LVOT VTI was our chosen reference standard for this study. The method suffers from poor performance in the hands of inexperienced operators. The study’s operators were trained using a supervised and standardised training programme based on current best practice to mitigate this effect.

## Conclusion

This study showed that there was poor agreement between the non-invasive methods used to measure SV/CO (bioreactance, PVUT CCABF, LVOT VTI and SSAD) and LVOT VTI in identifying fluid responders. Multiple studies have shown suboptimal agreement between methods used to assess stroke volume/cardiac output. Our data should not be interpreted as seeing one device as offering more accurate measurements than another but that there is limited agreement between the devices used in this study. Further studies are required to assess the accuracy of these non-invasive haemodynamic methods in spontaneously breathing patients in the ED prior to conducting large outcome trials based on their use.

## Data Availability

The datasets used and/or analysed during the current study are available from the corresponding author on reasonable request.

## Abbreviations

CCA: Common carotid artery
CCABF: Common carotid arterial blood flow
CNAP: Continuous non-invasive arterial pressure
ED: Emergency department
ICU: Intensive care units
IVC: Inferior vena cava
IVCCI: Inferior vena cava collapsibility index
LVOT VTI: Left ventricular outflow tract velocity time integral
PVUT: Plethysmography with vascular unloading technique
SBP: Systolic blood pressure
SSAD: Suprasternal aortic Doppler
USCOM: Ultrasound cardiac output monitor
